# Peptide-guided targeting of GPR55 for anti-cancer therapy

**DOI:** 10.18632/oncotarget.14121

**Published:** 2016-11-23

**Authors:** Maria Mangini, Enrico Iaccino, Maria Giovanna Mosca, Selena Mimmi, Rosa D’Angelo, Ileana Quinto, Giuseppe Scala, Stefania Mariggiò

**Affiliations:** ^1^ Institute of Protein Biochemistry, National Research Council, Naples, Italy; ^2^ Department of Experimental and Clinical Medicine, University ‘Magna Graecia’ of Catanzaro, Viale Europa, Località Germaneto, 88100 Catanzaro, Italy

**Keywords:** G-protein-coupled receptor (GPCR), GPR55, lysophosphatidylinositol (LPI), peptidic binder, phage-display screening

## Abstract

Expression of the lysophosphatidylinositol receptor GPR55 correlates with invasive potential of metastatic cells and bone metastasis formation of different types of tumors. These findings suggest a role for GPR55 signaling in cancer progression, including in lymphoproliferative diseases. Here, we screened a M13-phage-displayed random library using the bait of HEK293 cells that heterologously expressed full-length HA-GPR55. We selected a set of phagotopes that carried 7-mer insert peptides flanked by a pair of cysteine residues, which resulted in cyclized peptides. Sequencing of selected phagotopes dictated the primary structure for the synthetic FITC-labeled peptide P1, which was analyzed for binding specificity to immunoprecipitated HA-GPR55, and to endogenously expressed GPR55, using cells interfered or not for GPR55, as well as for co-localization imaging with HA-GPR55 at the membrane level. The peptide P1 stimulated GPR55 endocytosis and inhibited GPR55-dependent proliferation of EHEB and DeFew cells, two human B-lymphoblastoid cell lines. Our data support the potential therapeutic application of peptide ligands of GPR55 for targeting and inhibiting growth of neoplastic cells, which overexpress GPR55 and are dependent on GPR55 signaling for their proliferation.

## INTRODUCTION

G-protein-coupled receptors (GPCRs) are the largest family of membrane receptors in eukaryotes, and they mediate a wide variety of cellular responses through activation of heterotrimeric G-proteins [1]. G-proteins transduce the extracellular signals through a plethora of downstream effector molecules, such as adenylyl and guanylyl cyclases, phospholipases, phosphodiesterases, and ion channels, among others [2]. The pharmacological manipulation of GPCRs is a well-validated approach for human therapeutics in the cardiovascular, nervous, immune, metabolic, and endocrine systems [3]. Therefore, GPCRs represent attractive targets for novel therapeutic treatments of diseases that are correlated with up-regulated GPCR signaling, as in anti-cancer therapies. Indeed, correlations have emerged between GPCR expression levels and tumor development [4].

The lysophosphatidylinositol (LPI) receptor was recently identified as GPR55 [5], and based on the sequence similarities of GPR55 with rhodopsin, this 319-amino-acid protein belongs to the class A family of GPCRs [6]. Its lipid ligand, LPI, is produced from membrane phosphatidylinositol through the catalytic activity of Ca^2+^-dependent PLA_2_ or Ca^2+^-independent PLA_1_ [7]. LPI has pleiotropic activities in different cellular systems, and initial evidence of its role in cancer development was derived from clinical data, where LPI emerged as a marker of poor prognosis for patients with ovarian cancer [8]. This was in agreement with *in-vitro* studies that demonstrated high levels of lysophosphatidic acid, LPI, and their metabolites in tumor cells and transformed cells, as compared to their normal cell counterparts [7]. In some tumors, high extracellular levels of LPI and its metabolites have been observed due to decreased activity of the enzymes responsible for LPI catabolism [9, 10]. Indeed, signaling of lysophospholipid receptors is strongly reinforced in several tumors, as a consequence of receptor overexpression and/or increased availability of the relative ligands through their increased production or reduced degradation. In particular, Ras-transformed fibroblasts have a high intracellular content of LPI, which is secreted extracellularly and can stimulate cell proliferation in an autocrine manner [9].

Increasing evidence has also delineated the role of GPR55 in cancer development, as it is overexpressed in several tumor cells, including glioblastoma, astrocytoma, breast carcinoma, melanoma, ovarian carcinoma, B-cell multiple myeloma, and B-lymphoblastoid cells [5, 6, 11]. In particular, expression levels of GPR55 correlate with tumor aggressiveness [6]. Additional observations in GPR55 knock-out mice have indicated a role for GPR55 in bone metabolism. GPR55 is expressed in osteoblasts and osteoclasts, where LPI stimulates osteoclast polarization and bone resorption [6]. The evidence that LPI can be released from cancer cells suggests that GPR55 signaling can affect the tumor microenvironment and promote bone metastases [6]. Obtaining further insights into pharmacological manipulation of lysophospholipid metabolism and activation of lysophospholipid receptors and their downstream signaling should thus be relevant for development of novel approaches to cancer therapy.

The use of monoclonal antibodies for tumor immunotherapy is a valuable strategy for the targeting of tumor cells and to interfere with their neoplastic growth [12, 13]. In this context, GPR55 might represent an optimal target for cancer therapy. However, the lack of humanized monoclonal antibodies against GPR55 led us to develop peptide binders of this receptor for specific targeting of GPR55-positive tumor cells. Indeed, peptide binders of membrane receptors are an optimal tool for targeting neoplastic cells in the absence of antibody-based therapies [14]. As compared to monoclonal antibodies, peptides are less expensive, easier to manufacture and manipulate, and do not show batch-to-batch variations [15]. Moreover, peptides are not affected by the two main limitations of monoclonal antibodies: poor delivery to tumors due to their large size, and systemic toxicity due to non-specific uptake into the reticulo-endothelial system [16]. Peptides also have the advantage that they are smaller than antibodies and antibody fragments, and they show good tumor-penetrating activities and biocompatibility. Further, they do not bind to the reticulo-endothelial system, and do not elicit immune responses upon repeated administration [16]. As peptides can be degraded by proteases, they can be substituted with peptidomimetics that carry chemical modifications (e.g., cyclization, protection of the N-terminus and C-terminus), or non-natural amino acids, such as D-amino acids, which avoid protease-mediated degradation [17].

Here, we report on the identification and biological characterization of a peptide binder of GPR55 that specifically recognizes the receptor and inhibits the proliferation of EHEB and DeFew cells, two GPR55-positive B-lymphoblastoid cell lines.

## RESULTS

### Selection and characterization of peptide binders of GPR55

**Figure 1:**
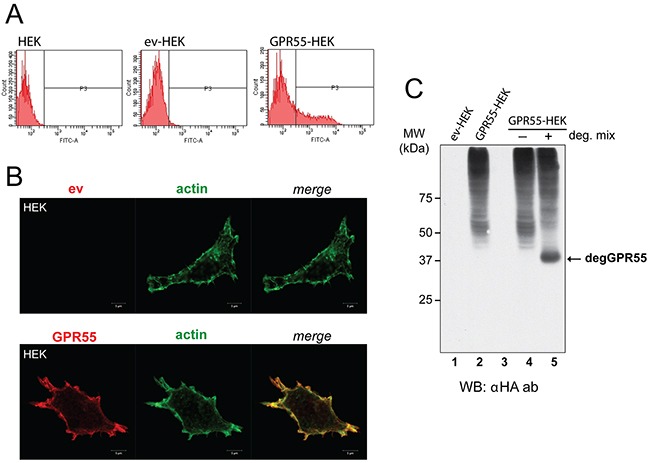
Characterization of cells overexpressing GPR55 as phage-display bait **A**. FACS analysis with anti-HA antibody of HEK293 cells untransfected (HEK) or transfected with the empty vector (ev-HEK), or HA-GPR55-expressing vector (GPR55-HEK). **B**. Representative images by confocal microscopy of ev-HEK and GPR55-HEK stained with anti-HA antibody and a secondary Alexa488-tagged anti-mouse antibody (red) for GPR55 staining, and Alexa546-phalloidin for filamentous actin (green). The two channels and their merge are shown. Scale bar: 5 μm. **C**. Membranes from ev-HEK (lane 1) or GPR55-HEK (lane 2) were left untreated or treated at 37°C without (−, lane 4) or with (+, lane 5) deglycosylating enzymes (deg. mix), and then analyzed by Western blotting with an anti-HA antibody (αHA ab). Lane 3 was left empty. MW, molecular weight standards. degGPR55, deglycosylated GPR55.

To identify peptide ligands of GPR55, the NEB C7C phage-displayed random peptide library was screened using as bait HEK293 cells that heterologously expressed HA-tagged GPR55. This approach allowed the native structure of this seven-transmembrane-domain receptor to be preserved. Supporting this experimental approach, whole-cell-based screening of ligands using peptide libraries has been successfully applied in reverse pharmacology to identify ligands of orphan receptors [18] and for the selection of ligands of other GPCRs [19]. HEK293 cells were chosen because they have been reported to lack expression of endogenous GPR55 [20]. Once transfected with the expression vector for HA-GPR55 [21], or the empty vector, the HEK293 cells were monitored for their GPR55 expression levels. Fluorescence-activated cell sorting (FACS) was performed with live cells, without any plasma membrane permeabilization, using an anti-HA antibody that in this way recognized only the extracellular HA epitope at the GPR55 N-terminus. FACS analysis indicated that 30% of HA-GPR55 transfected HEK293 cells showed plasma-membrane-localized receptor (Figure 1A, and Methods section). This GPR55 localization was confirmed in confocal images, where the anti-HA antibody showed the receptor in close proximity with cortical actin, which was decorated with fluorescent phalloidin, as part of the filamentous actin cytoskeleton (Figure 1B, and Supplementary Figure S1 for lower magnification).

Western blotting of membranes from these transfected HEK293 cells using an anti-HA antibody revealed a pattern of specific bands that also corresponded to the glycosylated forms of GPR55 (Figure 1C, lanes 2, 4). The treatment of cell membranes with a mix of deglycosylating enzymes resulted in the reduction of the slow-migrating bands, with concomitant appearance of a band that corresponded to the predicted 37-kDa deglycosylated GPR55 (Figure 1C, lane 5).

The selection of phage binders of GPR55 was obtained by screening a phage-displayed library. Briefly, the phages were precleared through incubation with wild-type HEK293 cells (Supplementary Figure S2, HEK, step 1), and empty-vector-transfected cells (Supplementary Figure S2, ev-HEK, step 2). The unbound phages were incubated with HEK293 cells expressing HA-tagged GPR55 (Supplementary Figure S2, GPR55-HEK, step 3). Non-interacting phages were washed out, while the plasma-membrane-bound phages and the internalized phages were collected (Supplementary Figure S2, step 4, extracellular and intracellular phage clones, respectively). A rapid acid wash with 100 mM glycine, pH 2.2, was used to detach the phages bound to the extracellular surface of the cells (Supplementary Figure S2, step 5), while the internalized phages were collected using cell cryolysis (Supplementary Figure S2, step 6). The separate pools of phages underwent additional rounds of selection; after each cycle, the phage colony-forming plaques were evaluated through *Escherichia coli* infection, which reached a plateau after four rounds of biopanning (Supplementary Figure S3A, S3B). Ultimately, 24 extracellular phage binders and 18 internalized phage binders were isolated and sequenced to determine the amino-acid sequence of the insert. Two amino-acid sequences (P1, P2) were identified in both the extracellular and intracellular phage clones, and one amino-acid sequence (P3) was selectively expressed in two extracellular phages (Table 1). Peptide P1 (CKKNSPTLC), which corresponded to the most recurrent insert among the phage binders, was selected for further characterization (Supplementary Figure S3C).

**Table 1:** Insert peptides and frequencies of the phage clones selected by screening the C7C phage-displayed random peptide library in the indicated cellular compartments

Peptide	Sequence (aa)	Number of isolated phage clones	
		Extracellular	Intracellular
P1	CKKNSPTLC	15/24	14/18
P2	CIGNSNTLC	7/24	4/18
P3	CAKATCPAC	2/24	0/18

### Validation of peptide P1 binding to GPR55

**Figure 2:**
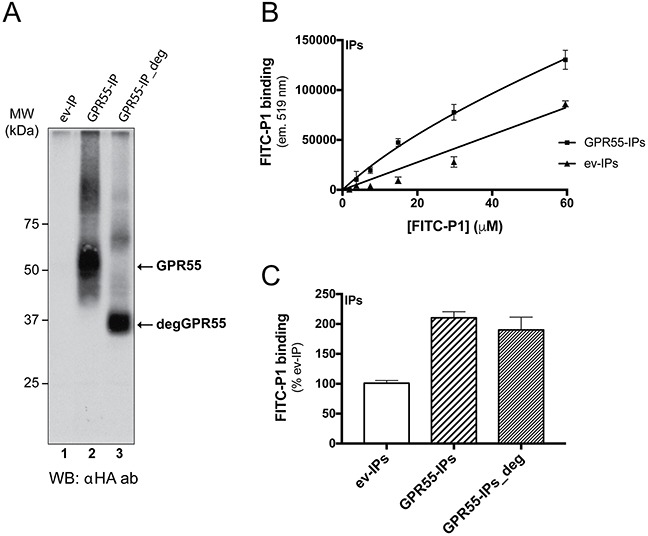
FITC-peptide-P1 binding to immunoprecipitated GPR55 **A**. Extracts of HeLa cells transfected with empty vector (ev) or HA-GPR55 (GPR55) were immunoprecipitated (IP) with purified anti-HA IgGs conjugated to Sepharose beads. IPs from cells transfected with HA-GPR55 were left untreated (GPR55-IP) or treated with a mix of deglycosylases (GPR55-IP_deg), and then analyzed by Western blotting using an anti-HA antibody (αHA ab). The arrows indicate the glycosylated GPR55 (GPR55) and the deglycosylated GPR55 (degGPR55) with the apparent molecular weight of 37 kDa. MW, molecular weight standards. **B**. Binding assays were performed with increasing concentrations of FITC-P1 with ev-IPs or GPR55-IPs for 2 h at 37°C. FITC-P1 binding was evaluated as FITC-fluorescence associated to the IPs (see Methods section). FITC-P1 binding affinity for GPR55 was in the range of 20 μM. Data are mean ±SD and are representative of three independent experiments (n = 3). **C**. Binding assays of FITC-P1 to glycosylated GPR55 and deglycosylated GPR55. The FITC-P1 peptide (40 μg/ml; 26.8 μM) was incubated with anti-HA-IPs (2.5 μl ev-IPs, GPR55-IPs or GPR55-IPs_deg) for 2 h at 37°C. FITC-P1 binding was evaluated as FITC-fluorescence associated to IPs, as percentage of ev-IPs fluorescence. Data are means ±SE of three independent experiments, each performed in triplicate.

Peptide P1 was synthesized and fluorescein-isothiocyanate (FITC)-conjugated at the N-terminus (FITC-P1), to determine whether it preserved its binding to GPR55 outside of the phage context. FITC-P1 binding was analyzed in immunoprecipitates obtained with an anti-HA antibody from cells transfected with pcDNA3 coding for HA-GPR55, or with the empty vector (Figure 2A). P1 showed concentration-dependent binding to both immunoprecipitates, with higher binding to HA-GPR55 immunoprecipitates (Figure 2B). From the P1-binding curves, an affinity binding constant of 20 μM was extrapolated, on the assumption that the binding obtained with immunoprecipitates from empty-vector-transfected cells was the non-specific binding, while the binding with HA-GPR55 immunoprecipitates was the total binding. We also verified the reversibility of FITC-P1 binding to HA-GPR55 immunoprecipitates, whereby a six-fold excess of unlabeled peptide P1, but not of an irrelevant peptide (Irr, see Supplementary Figure S3C, and Methods section), almost completely displaced the FITC-P1 bound to the receptor (data not shown).

As the overexpressed GPR55 is highly glycosylated (Figure 2A, lane 2), we determined whether receptor deglycosylation affected FITC-P1 binding to GPR55. As shown in Figure 2C, FITC-P1 bound equally to both glycosylated and deglycosylated GPR55-immunoprecipitates.

**Figure 3:**
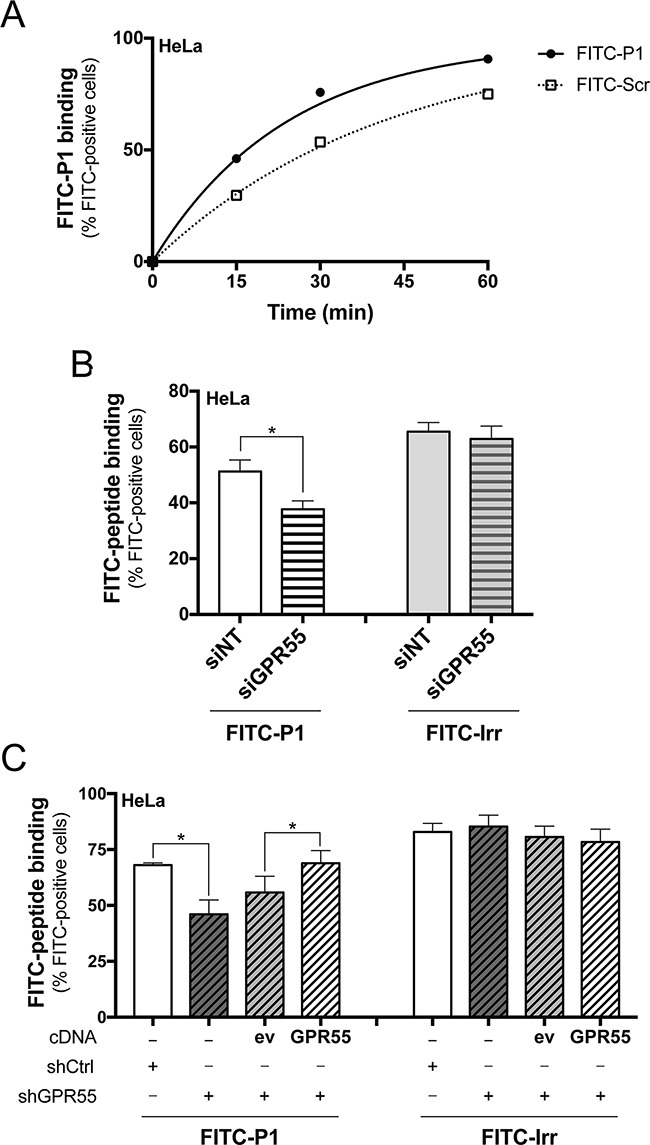
FITC-peptide-P1 binding to GPR55 in Hela cells **A**. Representative time-course of binding of FITC-P1 (40 μg/ml; 26.8 μM) and scrambled peptide (FITC-Scr, 40 μg/ml) to wild-type HeLa cells (150,000/6-well plate) at 37°C, with subsequent FACS quantification of cell-associated FITC fluorescence. Apparent K_d_ extrapolated was 20 μM. Peptide binding evaluated in subsequent FACS analysis of cell-associated FITC-fluorescence is shown, quantified as percentages of FITC-positive cells, and is representative of three independent experiments. **B**. FITC-P1 (40 μg/ml) or FITC-labeled irrelevant peptide (FITC-Irr, 40 μg/ml) were 1 h-incubated at 37°C with HeLa cells interfered with non-targeting (siNT), or with GPR55 siRNAs (siGPR55); peptide binding was subsequently evaluated by FACS analysis of cell-associated FITC fluorescence. Data are means ±SEM of four independent experiments. **C**. FITC-P1 (40 μg/ml) or FITC-Irr (40 μg/ml) were 1 h-incubated at 37°C with shCtrl, or shGPR55 without (−) or with empty-vector (ev), or HA-GPR55 transfection (GPR55); peptide binding was subsequently evaluated by FACS analysis of cell-associated FITC fluorescence. Data are means ±SEM of three independent experiments. *p <0.01 (Student's *t* tests).

In agreement with the immunoprecipitated GPR55, an apparent K_d_ of 20 μM was extrapolated from time-course analyses of FITC-P1 binding to intact HeLa cells, which endogenously express GPR55 (Figure 3A). In this assay, a FITC-labelled scrambled peptide (FITC-Scr, see Supplementary Figure S3C, and Methods section) showed lower binding to HeLa cells compared to FITC-P1 (Figure 3A). It is worth noting that peptide P1 was internalized in HeLa cells over 2 h of incubation at 37°C (as indicated by the addition of trypan blue; see Methods section), which will not have allowed peptide P1 binding to reach saturation levels for the dose-response curves (Supplementary Figure S4).

The selectivity of P1 binding to GPR55 was analyzed in HeLa cells under silencing for GPR55 with small-interfering (si)RNAs (siGPR55), compared to HeLa cells treated with non-targeting siRNAs (siNT). The efficiency of the siGPR55 was quantified by real-time PCR, which indicated 50% reduction in the basal GPR55 expression levels in siGPR55 HeLa cells. FACS analysis of HeLa cells incubated with FITC-P1 and a FITC-conjugated irrelevant peptide (FITC-Irr, see Methods section) indicated 26% decrease in P1 binding for siGPR55 relative to the control siNT, with the binding of the irrelevant peptide not significantly affected by siGPR55 (Figure 3B). This thus provided the first evidence that P1 binding parallels GPR55 expression, with higher binding to the siNT HeLa cells due to their higher expression of the receptor.

As the GPR55 silencing obtained with siRNAs was not optimal, we also selected HeLa cell clones that stably expressed a short hairpin (sh)RNA that was specific for human GPR55 (shGPR55), which showed 90% decrease in GPR55 mRNA expression (as evaluated by quantitative real-time PCR). In these clones, the re-expression of GPR55 was also possible by transient transfection of HA-GPR55, as the shGPR55 of choice targeted the non-coding region of the GPR55 gene, and thus did not interfere with the HA-GPR55-cDNA transfection. Cell clones that expressed a non-targeting shRNA (shCtrl) were also selected as the negative control. We then investigated FITC-P1 binding to these shGPR55 HeLa clones. FITC-P1 showed 34% less binding to shGPR55 HeLa clones relative to shCtrl, while the binding of the irrelevant peptide (FITC-Irr) was unaffected by shGPR55 (Figure 3C). Also GPR55 re-expression in shGPR55 HeLa clones recovered FITC-P1 binding, promoting a 25% increase in binding compared to the empty-vector-transfected shGPR55 HeLa clones (Figure 3C). Instead, GPR55 re-expression did not affect the interaction of the irrelevant peptide (Figure 3C), further indicating the specificity of P1 binding toward GPR55.

Altogether these data demonstrate that binding of peptide P1 to cells is dependent on the expression levels of GPR55.

**Figure 4:**
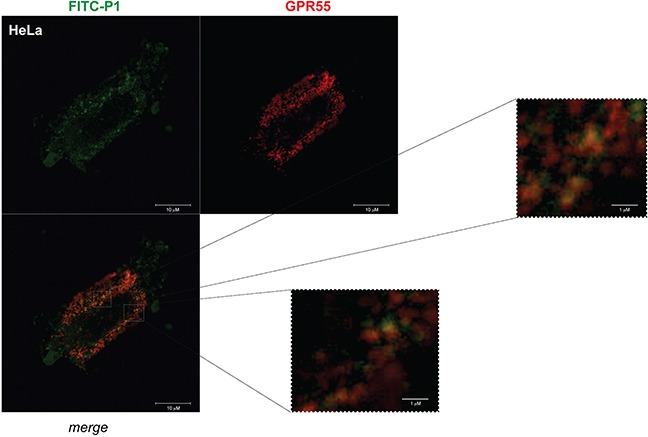
FITC-P1 co-localizes with HA-GPR55 Representative images from confocal analysis of HeLa cells overexpressing HA-GPR55 and incubated with FITC-P1. Live transfected cells were stained with the anti-HA antibody and secondary Alexa568-tagged anti-mouse antibody (red) for GPR55 staining, and then incubated for 1 h with 40 μg/ml (26.8 μM) FITC-P1 (green). After extensive washes, the cells were fixed and analyzed at the confocal microscope. The two channels and their merge are shown. Two magnifications of the boxed areas in the merge panel are shown. Scale bars: 10 μm (main panel); 1 μm (boxed areas).

The localization of P1 and GPR55 in HeLa cells was analyzed by confocal microscopy. We transfected HeLa cells with HA-GPR55 and monitored GPR55 expression with an anti-HA antibody. Both FITC-P1 and GPR55 were distributed at the membrane level, with evident spots of co-localization (Figure 4).

### Peptide P1 stimulates GPR55 internalization

**Figure 5:**
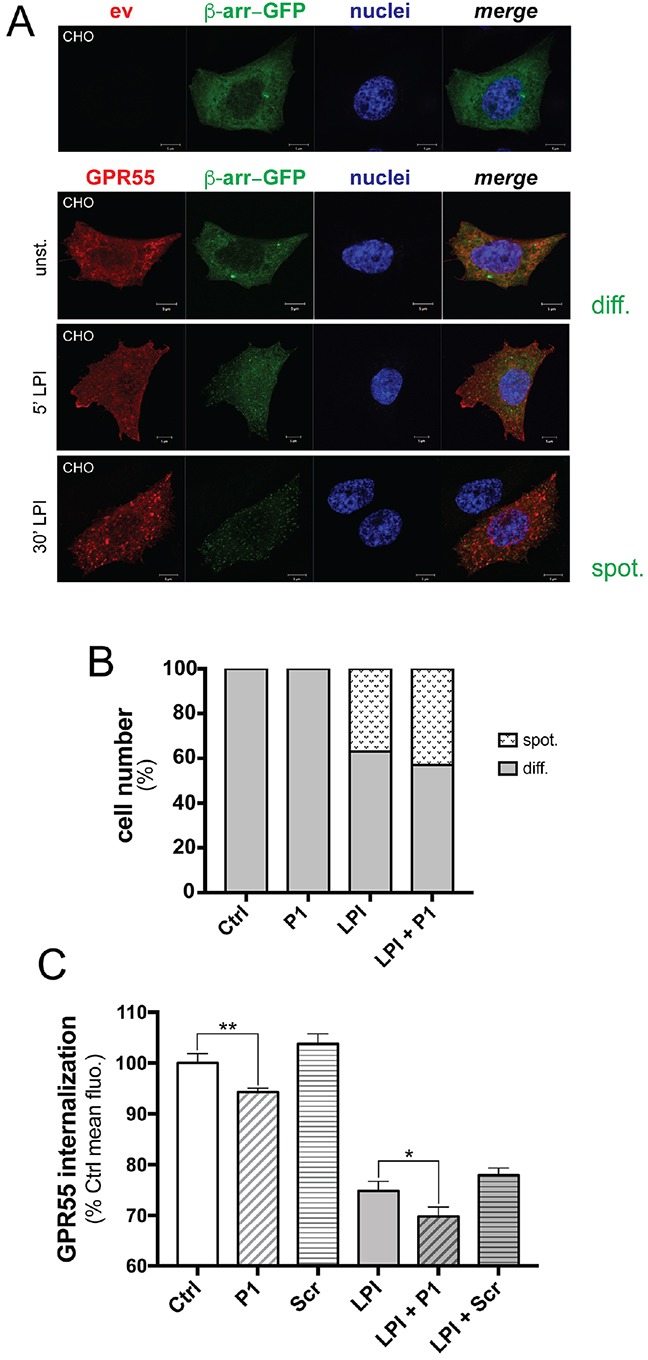
Peptide P1 stimulates GPR55 internalization **A**. Representative confocal images of CHO cells expressing empty vector (ev) or HA-GPR55E (GPR55, red) and β-arrestin–GFP (green). β-Arrestin–GFP was uniformly distributed in the cell cytoplasm (diff.) of unstimulated cells (unst.) after 1 h serum deprivation. In HA-GPR55E expressing cells, treatment for 5 min with 10 μM LPI caused plasma-membrane recruitment of β-arrestin–GFP, as shown by spotted distribution (spot.). Prolonged LPI treatment up to 30 min induced HA-GPR55E and β-arrestin–GFP co-localization in endocytic structures. Hoechst staining for cell nuclei (blue) is also shown. Scale bar: 5 μm. **B**. Quantification of the diffuse (diff.) and spotted (spot.) β-arrestin–GFP in CHO cells, as unstimulated, stimulated with LP1 (10 μM) for 30 min, without or with 10 min pre-incubation with peptide P1 (50 μg/ml). For each coverslip, 10 random fields were chosen blindly under a 40× objective, and the two opposite phenotypes were quantified; i.e. cells with β-arrestin diffuse in the cytoplasm (diff.), and with β-arrestin in vesicles (spot.). These two phenotypes were expressed as percentages of analyzed cells. Data are representative of three independent experiments. **C**. Quantification of plasma-membrane localization of HA-GPR55 in transfected HeLa cells by FACS analysis after immunostaining of live cells with the anti-HA and an Alexa488-tagged anti-mouse antibodies (see Methods section). Cells were pre-incubated or not with 80 μg/ml peptide P1 for 10 min, then stimulated or not with 10 μM LPI for 15 min. By FACS analysis, mean fluorescences were obtained, and compared to the mean fluorescence of the unstimulated sample (assumed as 100%), as indicative of decreased GPR55 plasma-membrane localization. The mean efficiency of transfection measured in these experiments was 58%, and the mean fluorescence of HA-GPR55 expressing HeLa cells was 5,800. Data are means ±SE of five independent experiments. *p <0.01, LPI+P1 *versus* LPI; **p <0.005, P1 *versus* unstimulated sample (Ctrl), (Student's *t* tests).

Following activation by their ligand, GPCRs are inactivated through kinase-dependent phosphorylation, which results in recruitment of the cytosolic protein β-arrestin, that then mediates internalization of the GPCR bound to its ligand, uncoupling the GPCR from its transduction machinery [22]. Thus, we asked whether peptide P1 can modulate spontaneous and agonist-induced internalization of GPR55. To test this hypothesis, we used Chinese hamster ovary (CHO) cells that co-expressed a functional chimera of β-arrestin–GFP and another HA-tagged GPR55 (HA-GPR55E, see Methods section). In the absence of GPR55 stimulation, β-arrestin–GFP showed diffuse cytosolic localization, as seen under confocal microscopy (Figure 5A, and Supplementary Figure S5 for lower magnification). Upon LPI treatment, the β-arrestin–GFP chimera translocated to the plasma membrane and induced HA-GPR55E internalization within 5 min (Figure 5A and Supplementary Figure S5), in a time-dependent manner. After 30 min of LPI stimulation, GPR55 endocytosis was observed in ~37% of the cells, and the β-arrestin–GFP chimera showed the characteristic spotted distribution, as seen under confocal microscopy (Figure 5A, 5B, and Supplementary Figure S5).

We then quantified these cells that showed the diffuse and spotted distribution of the β-arrestin–GFP chimera under the peptide treatment. Cell incubation with peptide P1 alone (50 μg/ml) did not affect the cytosolic distribution of β-arrestin (Figure 5B); however, pre-incubation with peptide P1 followed by LPI stimulation slightly increased agonist-induced GPR55 endocytosis, by 6.5%. The effects of P1 treatment on LPI stimulation were also observed under other assay conditions, with increased percentages of cells with the spotted distribution of the β-arrestin–GFP chimera ranging from 5% to 17%, depending on LPI concentration (1-10 μM), time of LPI stimulation (15-60 min), and P1 concentration (50-200 μg/ml) (data not shown).

The direct effects of peptide P1 on GPR55 localization at the membrane level were also analyzed by FACS in HeLa cells that expressed HA-GPR55, by staining with an anti-HA antibody without membrane permeabilization. The expression levels of HA-GPR55 at the plasma membrane were measured as the mean fluorescence of the transfected cells. As compared to unstimulated cells, LPI stimulation (10 μM LPI for 15 min) strongly decreased the membrane-associated signal of GPR55, by 24%, which indicated that there was GPR55 endocytosis (Figure 5C). Incubation of the cells with peptide P1 (80 μg/ml) decreased GPR55 localization at the plasma membrane by 6%, while a scrambled peptide slightly, but not significantly, increased the plasma membrane localization (Figure 5C). Pre-incubation of cells with peptide P1, but not with the scrambled peptide, increased LPI-induced GPR55 endocytosis by up to 30%, which suggested that a 6% increase in GPR55 internalization was promoted by peptide P1 (Figure 5C). The evidence of additive effects of LPI and peptide P1 on GPR55 internalization suggested that their relative molecular mechanisms are different. This hypothesis is supported by the evidence that peptide P1 had no significant effects on β-arrestin translocation to the membrane, which was indeed significantly induced by LPI. Thus, the mechanism of GPR55 endocytosis promoted by LPI appeared to be dependent on the β-arrestin pathway, while it was β-arrestin–independent in the case of peptide P1.

### Peptide P1 inhibits proliferation of B-lymphoblastoid cells

GPR55 triggering by ligands promotes activation of MAP kinases, which leads to cell proliferation [23]. To determine whether GPR55 signaling is affected by peptide-P1 binding, we analyzed cell proliferation and apoptosis induction/ sensitization in the absence and presence of peptide P1. As the GPR55 gene is highly expressed in cancer B cells [24], we performed this analysis in EHEB and DeFew cells, two B-lymphoblastoid cell lines.

Compared to HeLa cells, these EHEB and DeFew cells expressed 2.5-fold higher and similar levels, respectively, of GPR55 mRNA, as shown by real-time PCR. The effects of peptide P1 on cell proliferation were compared to those of O-1918 and ML-191, two specific and chemically distinct GPR55 antagonists. The treatment of EHEB cells with O-1918 (30 μM) and ML-191 (0.5 μM) resulted in 40% and 28% decreased cell numbers, respectively, after 96 h of culture, which indicated that GPR55 participates in the regulation of cell proliferation (Table 2). Similar treatments of DeFew cells resulted in decreased cell numbers of 38% with both O-1918 and ML-191, which indicated the similar participation of GPR55 in proliferation of DeFew cells (Table 2). Under the same experimental conditions, the cells were treated with peptides.

**Table 2:** Growth inhibition of B-lymphoblastoid cells by GPR55 antagonists

Cell type	Cell counts/ml (×1,000)		
	DMSO	O-1918(30 μM)	ML-191(0.5 μM)
EHEB	801 ±18	483 ±25*	578 ±49*
		(40%)	(28%)
DeFew	1053 ±41	648 ±10*	650 ±51*
		(38%)	(38%)

EHEB and DeFew cells were grown in the absence or presence of the indicated agents, which were added immediately after cell plating, and replenished every 24 h. The number of cells is reported after 96 h growth. Growth inhibition relatively to DMSO-treated cells is indicated in parentheses as % inhibition. Dead cells were <5% of total population, as measured by trypan blue-positive cells, independent of the sample. Data are means ±SEM of four independent experiments, each carried out in duplicate. * *p* <0.01 *versus* DMSO-treated cells (Student's *t* tests).

**Figure 6:**
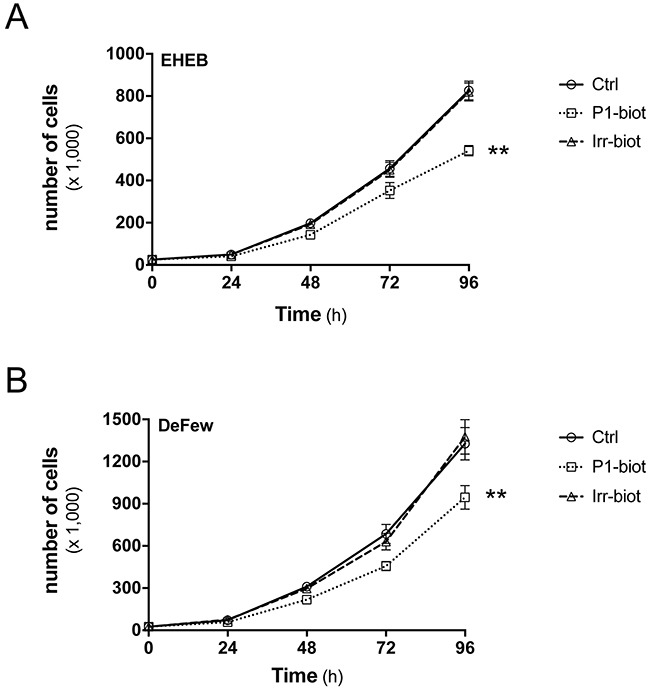
Peptide P1 inhibits B-lymphoblastoid cell proliferation Representative growth curves for EHEB **A**. and DeFew **B**. cells untreated (Ctrl) and treated with biotinylated peptide P1 (P1-biot, 1 μg/ml) or equimolar biotinylated irrelevant peptide (Irr-biot), added immediately after cell plating and every 24 h. Similar percentages of inhibition by biotinylated peptide P1 was observed up to 144 h of treatment in both cell lines. Data are means ±SEM of three independent experiments, each carried out in triplicate. **p <0.005 (Student's *t* tests).

To improve the peptide concentrations at the membrane level, biotinylated peptides were also synthesized and bound with streptavidin, to promote their oligomerization. In this proliferation assay, the biotinylated peptides were equipotent with the unmodified peptides (data not shown). With the EHEB and DeFew cells, biotinylated P1 reduced their numbers by 37% and 25%, respectively, while the biotinylated irrelevant peptide did not significantly affect cell proliferation (Figure 6). Differently from previous functional assays, due to the chronic treatment, the concentration of the peptide was lower (1 μg/ml). Figure 6 shows the effects produced by the highest effective concentration of biotinylated P1, with no effects seen for the ineffective equimolar biotinylated irrelevant peptide. Similar percentages of inhibition were observed up to 144 h of treatment (data not shown). This streptavidin-bound biotinylated P1 decreased cell numbers by 42% for EHEB cells and 34% for DeFew cells, thus reaching levels of inhibition that were similar to those of the GPR55 antagonists; in contrast, the streptavidin-bound biotinylated irrelevant peptide was poorly effective (Table 3).

**Table 3:** Growth inhibition of B-lymphoblastoid cells by peptides

Cell type	Cell counts/ml (×1,000)					
	Ctrl	P1-b	Irr-b	P1-b + str.	Irr-b + str.	Biotin + str.
EHEB	843 ±54	534 ±31**	830 ±53	486 ±29*	892 ±46	853 ±58
		(37%)	(2%)	(42%)	(−6%)	(−1%)
DeFew	1199 ±100	905 ±85**	1243 ±109	795 ±79*	1267 ±116	1209 ±107
		(25%)	(−4%)	(34%)	(−6%)	(−1%)

EHEB and DeFew cells were grown in the absence or presence of the indicated agents, which were added immediately after cell plating, and replenished every 24 h. The biotinylated peptide P1 (P1-b) was used at a concentration of 1 μg/ml (0.75 μM). The biotinylated irrelevant peptide (Irr-b), and biotin were added in equimolar amounts of P1-b. Streptavidin (str.) was used at 0.2 μM. The number of cells is reported after 96 h growth. Growth inhibition relative to untreated cells is indicated in parentheses as % inhibition. Dead cells were <5% of total population, as measured by trypan blue-positive cells, independent of the sample. Data are means ±SEM of four independent experiments, each carried out in duplicate. ** *p* <0.005 *versus* Ctrl; * *p* <0.01 *versus* cells treated with P1-b (one-way ANOVA, Tukey's multiple comparison test).

To dissect out the molecular mechanisms of peptide P1 inhibition of cell proliferation, we analyzed the cell-cycle distribution and appearance of apoptosis markers in EHEB cells treated with biotinylated peptides, or left untreated. The cell-cycle distribution indicated that treatment with biotinylated P1 or the biotinylated irrelevant peptides alone or oligomerized by streptavidin did not induce significant changes in the cell-cycle profile; similar results were obtained with the GPR55 antagonist ML-191 (0.5 μM) (Supplementary Figure S6).

Next, we analyzed whether peptide P1 counteracts cell growth through induction of apoptosis. As the externalization of phosphatidylserine on the plasma membrane of cells is a well-recognized marker of apoptosis, we measured EHEB cells that were positive for the binding of FITC-conjugated annexin V to phosphatidylserine, in the absence and presence of peptide P1. In FACS analysis, treatment with biotinylated P1 (alone or streptavidin-bound) did not increase annexin V cell decoration, similar to ML-191, and different from cells treated with the pro-apoptotic agent doxorubicin (10 μM) (Supplementary Figure S7). As an additional test of apoptosis, Western blotting of the cell extracts was used to evaluate pro-caspase 3 activation upon addition of ML-191, biotinylated P1, or the biotinylated irrelevant peptides (alone or streptavidin-bound), *per se* or in combination with a low concentration of doxorubicin (0.1 μM). Similar to ML-191 and the irrelevant peptide, biotinylated P1 (alone or streptavidin-bound) did not induce pro-caspase 3 activation, neither by itself, nor as potentiation of the doxorubicin treatment (Supplementary Figure S8).

These data indicated that peptide P1 inhibits cell proliferation of two B-lymphoblastoid cell lines in an equipotent manner to GPR55-specific antagonists without causing apoptosis.

## DISCUSSION

The *GPR55* gene is abundantly expressed in lymphoid organs, such as spleen and thymus, and hyper-expressed in many human cancer cells, including B-cell multiple myeloma, lymphoma, and lymphoblastoid cells [6, 23–25]. GPR55 expression levels correlate with tumor aggressiveness, and they are significantly elevated in high-grade tumors [6]. Increased expression levels of GPR55 can confer a proliferative advantage to cancer cells in cell culture and in tumor-xenograft mice, as a consequence of hyper-activation of the extracellular signal-regulated kinase cascade, one of the master regulators of cell proliferation [23]. GPR55 has also been shown to activate a key component of the pro-tumorigenic phosphoinositide 3-kinase/ Akt signaling pathway, while in IM-9 B-lymphoblastoid cells GPR55 regulates p38 activation [25]. GPR55 exerts these actions, at least in part, through the G_12/13_ and G_q_ proteins, which can signal oncogenic effects. In this regard, various studies have highlighted the role of G_12/13_ signaling in promoting cancer-cell proliferation, invasion, and metastatic spread through the activation of the Rho GTPases [21, 26, 27]. Altogether these findings suggest that GPR55 represents an intriguing target for the design of chemotherapeutic agents that can interfere with tumor growth.

Based on this hypothesis, the present study was designed to identify peptide ligands of GPR55 for targeting tumor cells that express this receptor, which would potentially be useful for diagnosis and therapy of proliferative diseases. A therapeutic effect might be derived upon peptide binding to GPR55 and the consequent modulation of its signaling, or upon targeted delivery to tumor cells of a chemotherapeutic agent carried by the peptide. We have previously applied this experimental approach to identify peptide binders of the B-cell receptor for antigen of human chronic lymphocytic leukemia [28], and murine A20 B-cell lymphoma [29, 30] for cancer monitoring and therapy.

Thus, we transfected HEK293 cells with HA-tagged GPR55 to have the bait for phage-display screening. A set of phage clones were isolated and characterized for their binding to GPR55-positive cells. The peptide P1 was synthesized based on the most recurrent primary sequence of the 7-mer peptide phage insert, which showed the highest binding to GPR55. Peptide P1 preserved binding and specificity towards immunoprecipitated GPR55, which indicated that it maintained the structural conformation of the peptide insert within the M13-phage coat protein. The binding of peptide P1 to GPR55 has a K_d_ of 20 μM, as extrapolated from binding assays with immunoprecipitated GPR55. These assays showed that peptide P1 binding was independent of GPR55 glycosylation status, which indicated that peptide P1 can target different cell systems that express GPR55, despite the variability of GPCR glycosylation within specific cellular contexts [31]. A similar binding affinity was calculated for peptide P1 toward intact HeLa cells that endogenously express GPR55. Loss of peptide P1 binding to intact HeLa cells was observed upon transient and stable GPR55 RNA interference with GPR55 siRNAs (siGPR55) or shGPR55, respectively. Recovery of peptide binding was then observed with GPR55 re-expression in the shGPR55 HeLa clones. These data indicated that peptide P1 binding to cells depends on the GPR55 expression levels.

Peptide P1 binding to GPR55 increased the rate of receptor internalization at steady-state as well as under LPI stimulation. GPR55 endocytosis induced by LPI contributes to the desensitization of the GPR55 signal. For GPCRs, this generally occurs through receptor phosphorylation (by a member of the family of GPCR kinases), β-arrestin recruitment, receptor binding to clathrin adaptor protein 2, and the consequent clustering into clathrin-coated pits for the receptor internalization [32]. Peptide P1 alone did not induce β-arrestin recruitment, even if it decreased plasma-membrane levels of GPR55 (by ~6%), suggesting that this GPR55 internalization was clathrin-independent and could follow alternative pathways, which would be caveolin dependent, or clathrin and caveolin independent [32].

Indeed, peptide P1 resembled an allosteric modulator, as it had an intrinsic activity and the potential to modulate orthosteric ligand effects [33]. Consistent with this hypothesis, the effects of LPI and peptide P1 on GPR55 internalization were additive. Allosteric ligands have been shown to modulate GPCR regulatory processes, including receptor expression levels on the plasma membrane, through induction of internalization, or conversely, by increasing the cell-surface expression [34]. Examples of allosteric modulators that can induce GPCR endocytosis with potential pharmacological applications include: the muscarinic acetylcholine receptor modulator LY2033298 [35]; the cannabinoid CB1 ligand Org27569 [36]; the small-molecule allosteric agonist of glucagon-like peptide1-receptor Compound 2 [37]; and AMN082, which promotes internalization of the glutamate receptor mGluR7 [38].

The therapeutic relevance of these modulators relies on their functional antagonism, whereby internalization of the receptor inhibits its function by simply removing it from the cell surface [34]. This led us to speculate that peptide P1 binding to GPR55 might inhibit receptor signaling by decreasing the GPR55 levels at the plasma membrane, or by modulating GPR55 coupling with the transduction machinery. These hypotheses were tested in two B-lymphoblastoid cell lines, EHEB and DeFew cells, the proliferation of which partially depends on GPR55 signaling. This was shown by their inhibition of proliferation (up to 40%) upon treatment with two distinct and specific GPR55 antagonists. In both of these tumor cells, peptide P1 induced inhibition of cell growth without causing apoptosis. The peptide P1 effects were reinforced by streptavidin-mediated oligomerization of biotinylated P1, as a consequence of increased P1 concentration at the plasma membrane, or by forced GPR55 oligomerization induced by oligomerized P1 binding, and the consequent interference with steady-state receptor cooperation and cell-signal integration [39, 40]. Streptavidin-conjugated peptide P1 showed inhibition of cell proliferation that was almost equipotent to the GPR55 antagonists, indicating that it likley blocked the fraction of cell proliferation that was dependent on GPR55 signaling.

Peptide binders of GPR55 might represent a valuable tool for *in-vivo* monitoring of tumor cells and tumor-specific delivery of chemotherapeutic agents. To this end, further investigations on the biological effects of such GPR55 peptide binders for direct modulation of GPR55 signaling are required. Peptide binders represent a feasible approach for tumor therapy, and their use might go beyond active immunotherapy and vaccination [41], with interesting applications for targeted and combination therapies [16, 42].

## MATERIALS AND METHODS

### Materials

Dulbecco's modified Eagle's medium (DMEM), minimum essential medium (MEM), Roswell Park Memorial Institute (RPMI) 1640 medium, fetal bovine serum (FBS), nonessential amino acids, Hanks balanced salt solution with calcium and magnesium (HBSS^++^), and phosphate-buffered saline (PBS) were from Gibco (Life Technologies Italia, Italy). Penicillin-streptomycin, L-glutamine, nonfat milk, L-α-lysophosphatidylinositol sodium salt from *Glycine max* (soybean), bovine serum albumin (BSA), fatty-acid-free BSA, Tween-20, biotin, propidium iodide, and 0.4% trypan blue solution in PBS were from Sigma-Aldrich (St. Louis, MO, USA). Puromycin, ML-191, and O-1918 were from Calbiochem (San Diego, CA, USA). Lipofectamine 2000, Alexa568, and Alexa488-tagged anti-mouse secondary antibodies were from Invitrogen (Carlsbad, CA, USA). All of the synthetic peptides were from CASLO ApS (Lyngby, Denmark). Based on the sequence of peptide P1 (CKKNSPTLC), both a scrambled peptide (KCLTSNCPK), with the same amino-acid composition as P1 but a different primary sequence, and an irrelevant peptide (CGGNGPGLC) were designed. The irrelevant peptide had the same length as P1, but included mutations to all of the polar amino acids of peptide P1, as these might represent ‘sticky’ residues (shown in the blue letter code of the amino-acid residues in Supplementary Figure S3C), and to some of the residues conserved between peptide P1 and P2, as these might be relevant for GPR55 recognition and binding (shown in the red letter code of the amino-acid residues in Supplementary Figure S3C). Peptide P1, the scrambled and the irrelevant peptides were cyclized using an intramolecular disulfide bond between the two cysteine residues. The fluorescent peptides were obtained by conjugation at the N-terminus with FITC *via* an aminohexanoic acid linker, while the biotinylated peptides were obtained by conjugation at the N-terminus with biotin *via* an aminohexanoic acid linker.

### Cell culture and growth curves

HEK293 cells were bought in 2012, from the American Type Culture Collection (ATCC catalogue number: CRL-11268), and were grown in monolayers in DMEM supplemented with 10% FBS, 2 mM L-glutamine, 100 U/ml penicillin, and 100 μg/ml streptomycin. HeLa cells were received from Dr. D. Corda, Institute of Protein Biochemistry, CNR, Naples, which were bought in 2006 from the European Collection of Cell Culture (ECACC catalog number: 93021013). Wild-type HeLa cells were maintained in MEM with 10% FBS, 2 mM L-glutamine, 100 U/ml penicillin, 100 μg/ml streptomycin, and nonessential amino acids (i.e., 100 μM glycine, L-alanine, L-asparagine, L-aspartic acid, L-glutamic acid, L-proline, L-serine). The HeLa clones were selected in the presence of 0.3 μg/ml puromycin. CHO cells were received from Dr. D. Corda [43], and were grown in DMEM supplemented with 10% FBS, 2 mM L-glutamine, 100 U/ml penicillin, 100 μg/ml streptomycin, and nonessential amino acids. EHEB cells are an Epstein-Barr-virus-immortalized cell line that was established from a chronic lymphocytic leukemia patient [44, 45], and these were purchased from DSMZ (Deutsche Sammlung von Mikroorganismen und Zellkulturen GmbH, Braunschweig, Germany). DeFew cells are a non-Hodgkin lymphoma cell line, and these were obtained from Prof. G. Scala, ‘Magna Graecia’ University of Catanzaro [46–48]. Both of these human B-lymphoblastoid cell lines were maintained in RPMI 1640 with 10% heat-inactivated (30 min at 55°C) FBS, 2 mM L-glutamine, 100 U/ml penicillin, and 100 μg/ml streptomycin, and were routinely tested for BCR expression and anti-IgG stimulation, according to previous studies [45–48]. All of the cells were grown in a humidified atmosphere of 5% CO_2_ and at 37°C return.

The EHEB cell growth rate was evaluated by cell counting. EHEB and DeFew cells were plated into 24-well plates in 400 μl growth medium at a density of 25,000 cells/ml. The cells were grown in the absence and presence of different agents, which were added immediately after cell plating and replenished every 24 h. Every 24 h, the cells were put through independent and blinded counts in triplicate, using a Neubauer cell-counting chamber. After 48 h, the cells were diluted 1:2 with fresh medium and 400 μl were transferred in a new 24-well for the following peptide additions. At 96 h, the cells were counted with 0.2% trypan blue, to determine cell viability.

### Transfection and RNA interference

For phage-display, the day before transfection, HEK293 cells to be used as bait were plated at 5 ×10^6^ cells/100-mm Petri dish in growth medium without antibiotics, and transfected with 15 μg pcDNA3 expressing human HA-GPR55 (from Dr. K. Mackie, Indiana University, Bloomington, IN, USA) [21, 26, 27], or the empty vector, using Lipofectamine 2000, according to the manufacturer instructions.

For the immunoprecipitation assays, HeLa cells were plated at 900,000 cells/100-mm Petri dish in growth medium without antibiotics. Twenty-four hours later, cell dishes were transfected with 10 μg pcDNA3.1 (ev) or with ss-3×HA-hGPR55HA in pcDNA3 (HA-GPR55), which has a triple HA tag at the N-terminus and an optimized signal sequence (ss, from amino acids 1-33: MATGSPTSLLLAFGLLCLPWLQEGSARDPPVAT, derived from human growth hormone) for efficient plasma-membrane localization (from Dr. A. Irving, Dundee University, UK) [21, 26, 27], using Lipofectamine 2000, according to the manufacturer instructions.

For transient interference of GPR55, HeLa cells were plated at 150,000 cells/6-well plate in growth medium without antibiotics, and 24 h later they were transfected with 75 pmol/well non-targeting #2 siRNA (siNT) or human GPR55-targeting siRNAs (D-00581-05) (siGPR55) (siGENOME, Dharmacon, Chicago, IL, USA), using Lipofectamine 2000, according to the manufacturer instructions. Twenty-four hours later, the cells were detached by trypsinization, plated again at 150,000 cells/6-well plate, and 10 h later they were re-transfected with siNT or with siGPR55. Forty-eight hours later, the cells were analyzed. For stable interference of GPR55, HeLa cells were transfected with 1.7 μg/well OmicsLink shRNA expression clone CSHCTR001-CU6 (shCtrl) or clone HSH022476-3-CU6 (shGPR55) from GeneCopoeia (Rockville, MD, USA), using Lipofectamine 2000, according to the manufacturer instructions. Forty-eight hours after transfection, HeLa clones stably expressing shRNAs were selected in growth medium containing 0.3 μg/ml puromycin.

### RT-PCR and real-time PCR

Total RNAs were extracted using RNeasy kits (Qiagen, Hilden, Germany), cDNAs were obtained with QuantiTect Reverse Transcription kits (Quiagen), PCRs were performed using Taq DNA polymerase (Life Technologies, CA, USA), and real-time PCRs with QuantiTect SYBR Green PCR kits (Qiagen), according to the manufacturer instructions. The primers used for both PCR and real-time PCR were: GPR55 forward, 5′-TCTACATGATCAACCTGGCAGTCT-3′; GPR55 reverse, 5′-CTGGGACAGGACCATCTTGAA-3′. For the housekeeping gene followed as the control, human hypoxanthine phosphoribosyltransferase (HPRT1), the primers were: HPRT1 forward, 5′-TGCTGACCTGC TGGATTACA-3′; HPRT1 reverse, 5′-CCTGACCAAGG AAAGCAAAG-3′. The real-time PCR program consisted of an initial 15 min at 95°C, and then 45 cycles as the following: 94°C for 15 s, 52°C (GPR55 primers) or 60°C (HPRT1 primers) for 30 s, and 72°C for 30 s. The PCR machine used was a Rotor Gene Q (Qiagen).

### GPR55 quantification by FACS

Twenty-four hours after transfection, HEK293 cells were stained with an anti-HA antibody (1:1,000) in PBS plus 3% BSA, on ice for 1 h, washed three times with cold PBS, incubated with an Alexa488-tagged anti-mouse antibody (1:800) in PBS plus 3% BSA for 45 min on ice. After three washes in cold PBS, the cells were incubated for 5 min at 37°C with PBS plus 2 mM EDTA, and detached by scraping. Then, the cells were centrifuged at 300× *g*, resuspended in PBS plus 3% BSA, and analyzed by FACS.

For the GPR55 internalization assay, HeLa cells at 24 h after transfection were serum deprived for 2 h in MEM plus 2 mM glutamine, and incubated with 80 μg/ml peptide P1 or the irrelevant peptide, in 1 ml HBSS^++^ plus 0.01% fatty-acid-free BSA, at 37°C for 10 min. Then 10 μM LPI was added for a further 15 min, and incubations were terminated by cell washing with cold PBS, twice. The cells were then stained and analyzed as reported for HEK293 cells.

### Immunofluorescence microscopy

HEK293 or CHO cells were rinsed with PBS, fixed for 10 min at room temperature in 4% (v/v) paraformaldehyde, and permeabilized in 0.02% saponin, 0.5% BSA, and 50 mM ammonium chloride in PBS (blocking solution) [49]. Then, the cells were stained with an anti-HA antibody (1:1,000) for 1 h at room temperature for GPR55 visualization, followed by 30 min at room temperature with Alexa488- or Alexa568-conjugated anti-mouse secondary antibodies (1:800), with 33 nM Alexa546-labeled phalloidin (Life Technologies) for filamentous actin visualization, or 2 μg/ml Hoechst (Sigma) for nuclei staining, with all reagents diluted in blocking solution. Then, the cells were washed three times with PBS plus 0.02% Tween-20, and the coverslips were mounted and examined by confocal microscope (LSM 510; Zeiss, Germany), with images acquired using the ZEN 2.1 (black) program (Carl Zeiss Microscopy GmbH 1997-2015). For localization studies of FITC-P1 and GPR55 in live HeLa cells, the cells were incubated with an anti-HA antibody (1:1,000) in PBS plus 3% BSA for 1 h on ice, then washed with PBS, and incubated with an Alexa568-conjugated anti-mouse secondary antibody (1:800) in PBS plus 3% BSA for 45 min on ice. The cells were then incubated with FITC-P1 as reported, fixed for 10 min in 4% (v/v) paraformaldehyde, and examined by confocal microscopy, as above.

### Western blotting

Cell lysates were obtained by scraping the cells into RIPA buffer: 50 mM Tris-HCl, pH 7.5, 300 mM NaCl, 1% sodium deoxycholate, 1% Triton X-100, 0.1% sodium dodecyl sulfate, plus protease inhibitors (Roche, Indianapolis, IN, USA). Following gentle homogenization by 20 passages through a 26-gauge needle, the lysates were centrifuged at 10,000× *g* for 5 min at 4°C, and the supernatants were collected. One hundred micrograms of these supernatants were subjected to SDS-PAGE, and transferred to nitrocellulose membranes (PerkinElmer Life Science, Boston, MA, USA). For immunoblotting, the membranes were blocked with 5% nonfat milk in TBS (10 mM Tris-HCl, pH 7.4, 10 mM NaCl) plus 0.1% Tween-20 (T-TBS) for 30 min at room temperature, and incubated with primary antibodies in T-TBS plus 3% BSA for 2 h at room temperature or overnight at 4°C. The membranes were washed twice in T-TBS for 7 min, and then incubated with secondary antibodies conjugated to horseradish peroxidase (1:5,000) (Calbiochem, San Diego, CA, USA) in T-TBS with 5% nonfat milk for 30 min at room temperature. The membranes were then washed twice with T-TBS and once with TBS for 5 min, and the signals were detected by ECL (Amersham Pharmacia, Piscataway, NJ, USA).

The mouse monoclonal anti-HA (16B12) antibody (dilution, 1:1,000) was from Covance, and the rabbit polyclonal anti-caspase 3 (H-277) antibody (dilution, 1:1,000) was from Santa Cruz Biotechnology (San Diego, CA, USA).

### Selection and amplification of GPR55-binding phages

The NEB C7C phage-displayed random peptide library had a diversity of about 10^9^ phage clones. The insert peptides from this library consisted of sequences of seven amino acids that were flanked by two cysteine residues. This resulted in a phage display of cyclized peptides expressed on the gene-3 minor coat protein of M13 phage [29]. The selection procedure was according to previous reports [29, 50]. Briefly, phages of approximately 1 ×10^10^ plaque-forming units (PFU) were pre-incubated for 1 h with 5 ×10^5^ wild-type HEK293 cells. The supernatant containing unbound phage particles was subsequently transferred to 35-mm Petri dishes to be incubated with 5 ×10^5^ ev-HEK293 cells for an additional 2 h, to further eliminate phage particles with aspecific binding. The ultimate pre-cleaned supernatant was incubated with GPR55-HEK293 cells for 2 h, and unbound phage particles were removed by washing with 50 mM Tris-HCl, pH 7.5, 150 mM NaCl, 0.1% Tween-20. The extracellular-bound phages were eluted with 100 mM glycine-HCl (pH 2.2) for 10 min, and neutralized with 1 M Tris-HCl (pH 9.0). An additional step of cryogenic lysis of GPR55-HEK293 cells allowed the recovery of the intracellular-bound phages. Extracellular- and intracellular-bound enriched-phages were then separately amplified by infecting *Escherichia coli* XL1-blue bacteria, and then purified using polyethylene glycol with four rounds of selection using GPR55-HEK293 cells as bait.

### Immunoprecipitation

Twenty-four hours after transfection, HeLa cells were washed three times with cold PBS and scraped into RIPA buffer (50 mM Tris-HCl, pH 7.5, 300 mM NaCl, 1% sodium deoxycholate, 1% Triton X-100, 0.1% sodium dodecyl sulfate) containing protease inhibitors (Roche, Indianapolis, IN, USA). Following gentle homogenization by passage through a 26-gauge needle, the lysates were centrifuged at 1,800× *g* for 5 min at 4°C, and the supernatants were collected and quantified for protein content. One mg of supernatant protein was subjected to immunoprecipitation using 10 μl Anti-HA.11 Epitope Tag Affinity Matrix (2 mg/ml) (Covance, Princeton, NJ, USA), for 16 h at 4°C. The immunoprecipitates (IPs) were washed twice with cold RIPA buffer and once with cold PBS, resuspended in PBS, and used as bait to test peptide binding.

### Deglycosylation

Deglycosylation reactions were performed starting from 100 μg HEK293 cell membranes, or IPs obtained from 100-mm Petri dishes of HeLa cells. For membrane preparation, HEK293 cells were lysed in 10 mM Tris-HCl, pH 7.5, 5 mM EDTA, 7 mM MgCl_2_, containing protease inhibitors (Roche), using a Polytron (UltraTurrax) homogenizer. Postnuclear supernatants prepared by centrifugation (500× *g* for 10 min at 4°C) were further centrifuged at 13,500× *g* for 30 min at 4°C, and the resulting pellets were resuspended in 10 mM Tris-HCl, pH 7.5, 150 mM NaCl, 25 mM KCl, 1 mM CaCl_2_, 0.3% Triton X-100. Cell membranes or IPs were incubated with 10 μl or 1 μl Protein Deglycosylation Mix (New England Biolabs, Hitchin, UK), respectively, for 4 h at 37°C. The incubations were terminated by addition of Laemmli sample buffer, and the reactions were subjected to SDS-PAGE and Western blotting.

### Peptide binding to immunoprecipitated GPR55

One milligram of cell proteins obtained from HeLa cells transfected with empty-vector (ev) or HA-GPR55 (GPR55) was immunoprecipitated (IP) with 10 μl anti-HA.11 Epitope Tag Affinity Matrix (2 mg/ml) in RIPA buffer, as detailed above. After PBS resuspension, 2.5 μl ev-IP or GPR55-IP were incubated with increasing concentrations (2.5, 5, 10, 20, 40 μg/ml) of FITC-P1 in HBSS^++^ plus 0.1% fatty-acid-free BSA, for 2 h at 37°C, with gentle agitation. After incubation, the IPs were washed four times in PBS plus 0.1% Tween-20, once in PBS, re-suspended in 40 μl PBS, and transferred in a 96-well plate. The FITC-fluorescence associated with the beads was quantified by spectrofluorimetric analysis (excitation, 495 nm; emission, 519 nm) (Multimode Plate Reader, Enspire, Perkin Elmer).

### Peptide binding to intact cells

Wild-type HeLa cells plated at a density of 150,000 cells/6-well plate, or cells stable interfered with shCtrl and shGPR55 plated at a density of 120,000 cells/6-well plate, or HeLa cells transiently interfered (48 h after the first interference) were washed twice with HBSS^++^, and incubated with the indicated concentrations of the FITC-labeled peptides for the indicated times at 37°C in 1 ml HBSS^++^ plus 0.1% fatty-acid-free BSA and 25 mM HEPES, pH 7.5. Then, the cells were washed twice with PBS plus 0.01% Tween-20, and once with PBS, and incubated for 5 min at 37°C with PBS with 2 mM EDTA, detached by scraping, and resuspended in PBS plus 3% BSA. Cell-associated FITC fluorescence was evaluated using FACS, after addition of 4 μg/ml propidium iodide, to exclude dead cells from the FACS gating. Fluorescence intensity was evaluated as % FITC-positive cells and mean fluorescence, recorded before (for peptide binding) and after (for peptide internalization) addition of 0.2% trypan blue to quench the fluorescence of the extracellular bound peptides.

### β-arrestin assay

CHO cells were plated (100,000 cells/24-well plate) on coverslips in growth medium without antibiotics. Twenty-four hours later, the cells were transiently transfected with expression vectors: human 1xHA-GPR55E in pCDNA3.1zeo(−) (0.5 μg, HA-GPR55E) and β-arrestin–GFP in pEGFP vector (70 ng). HA-GPR55E is the cDNA of the human GPR55 with the vasopressin receptor-2 C-tail (HRPSRVQLVLQDTTISRG), from the Addiction Research GPCR assay bank (Duke University, Durham NC, USA). The GPR55 vector was engineered to have more efficient β-arrestin coupling, without affecting the LPI–receptor interactions [51]. Twenty-four hours after transfection, the cells were washed with HBSS^++^ before incubation in HBSS^++^ plus 0.01% fatty-acid-free BSA. Agonist-stimulated redistribution of β-arrestin–GFP was assessed for up to 60 min of LPI treatment at 37°C. The cells were then fixed with 4% (v/v) paraformaldehyde for 10 min at room temperature, followed by anti-HA and Hoechst staining (see above), and analyzed by confocal microscopy. Using a 40× objective, 10 random fields were chosen blindly and analyzed per coverslip, and the number of all of the transfected cells were analyzed by counting the cells with the two opposite phenotypes, i.e., cells with β-arrestin diffuse in the cytoplasm (diff.) or with β-arrestin in vesicles (spot.). The two phenotypes were expressed as percentages of the analyzed cells.

### Cell-cycle analysis and annexin-V staining

To evaluate the cell-cycle distribution, the EHEB cells were treated as described for the growth-curve evaluation. At the end, 96 h after plating, 1 ×10^6^ cells were harvested, washed twice with cold PBS, and centrifuged at 300× *g* for 10 min. The cell pellet was gently resuspended in 1 ml cold 70% ethanol, added dropwise, and incubated at 4°C for 48 h. The cells were centrifuged again at 300× *g* for 10 min, washed, and resuspended in PBS plus 100 μg/ml RNAse A (Invitrogen), and incubated at 37°C for 30 min. After this incubation, 20 μg/ml propidium iodide was added, and the cells were further incubated on ice in the dark for 30 min, and finally analyzed by FACS.

In parallel, for analysis of apoptosis markers, 200,000 cells/sample were harvested, washed in PBS, stained with fluorescent annexin V using human annexin V-FITC kits (Bender MedSystems GmbH, Vienna, Austria), according to the manufacturer instructions, and analyzed by FACS.

## SUPPLEMENTARY FIGURES


