# Supplementary material for: Peptide-guided targeting of GPR55 for anti-cancer therapy
Source: Oncotarget. 2016 Nov 23;8(3):5179–95. doi: 10.18632/oncotarget.14121 (PMC5354900; doi:10.18632/oncotarget.14121)
Supplement: Supplementary file 1 [file oncotarget-08-5179-s001.pdf]

Peptide-guided targeting of GPR55 for anti-cancer therapy

SUPPLEMENTARY FIGURES

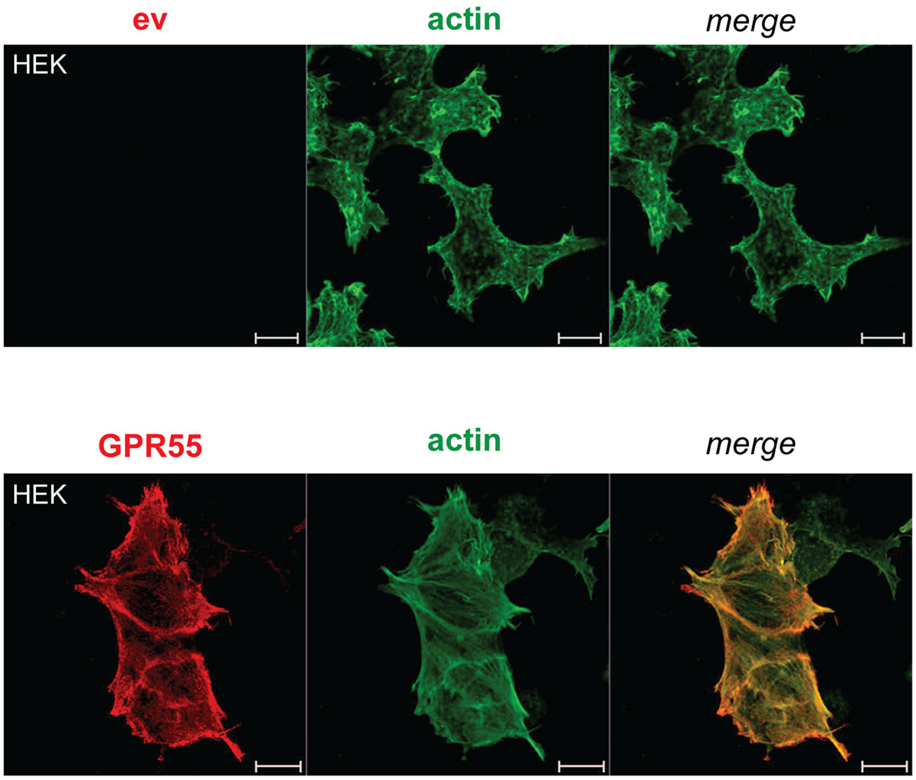

Supplementary Figure S1: Immunofluorescence analysis of transfected HEK293 cells. Representative images by confocal microscopy of HEK293 cells transfected with the empty vector (ev) or HA-GPR55-expressing vector (GPR55), stained with the anti-HA antibody and a secondary Alexa488-tagged anti-mouse antibody (red) for GPR55, and Alexa546-phalloidin for filamentous actin (green). The two channels and their merge are shown. Scale bar: 10 µm.

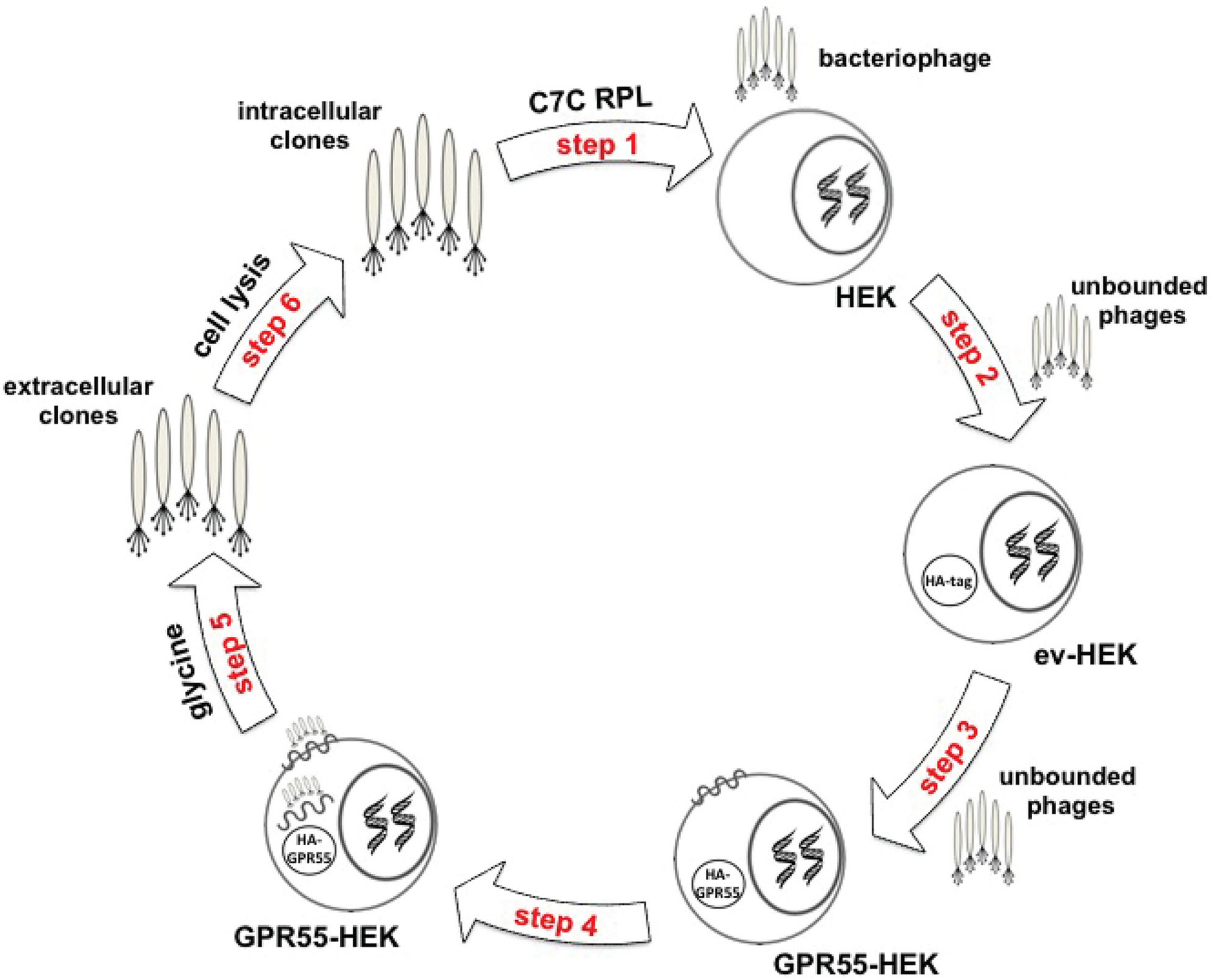

Supplementary Figure S2: Phage-display screening. Scheme of the screening for GPR55-phage binders. C7C RPL, NEB C7C random peptide library.

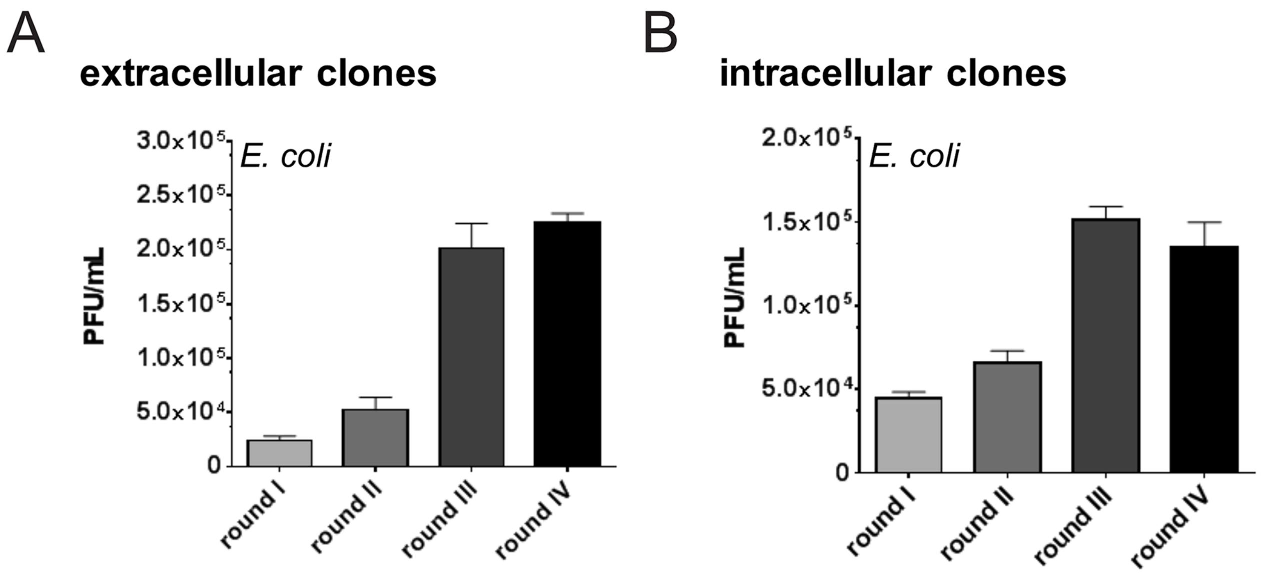

C

mimotope 1 (P1)	C KKNSPTLC
mimotope 2 (P2)	C I GNSNTLC
irrelevant peptide (Irr)	C GGNGPGLC
scrambled peptide (Scr)	KCLTSNCPK

Supplementary Figure S3: GPR55 phage-display screening. Colony-forming capacity of **A.** plasma-membrane bound (extracellular) clones, and **B.** internalized (intracellular) clones, after sequential cycles of screening. **C.** Sequences of peptides P1 and P2, irrelevant peptide (Irr), and scrambled peptide (Scr), (see Methods section).

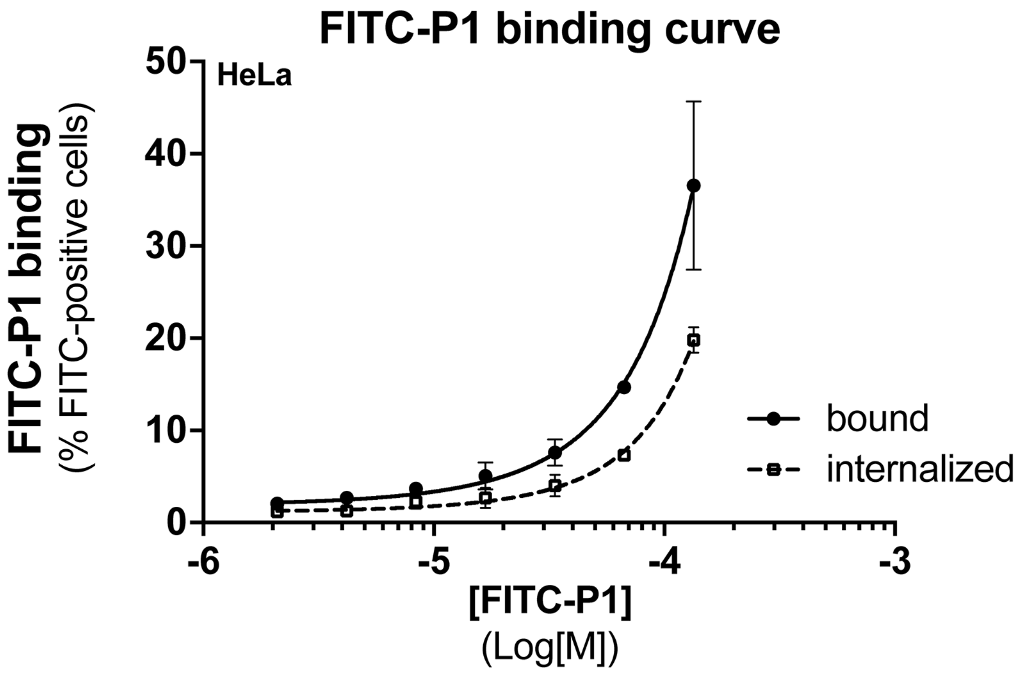

Supplementary Figure S4: FITC-P1 binding to GPR55. Binding of increasing concentrations of FITC-P1 in HeLa cells for 2 h at 37 °C. The FITC-P1 binding was evaluated in subsequent FACS analysis of cell-associated FITC-fluorescence (as percentages of FITC-positive cells). Data are means ±S.D. of three independent experiments. After the first analysis, trypan-blue addition provided quantification of internalized peptides (dotted line), (see Methods section).

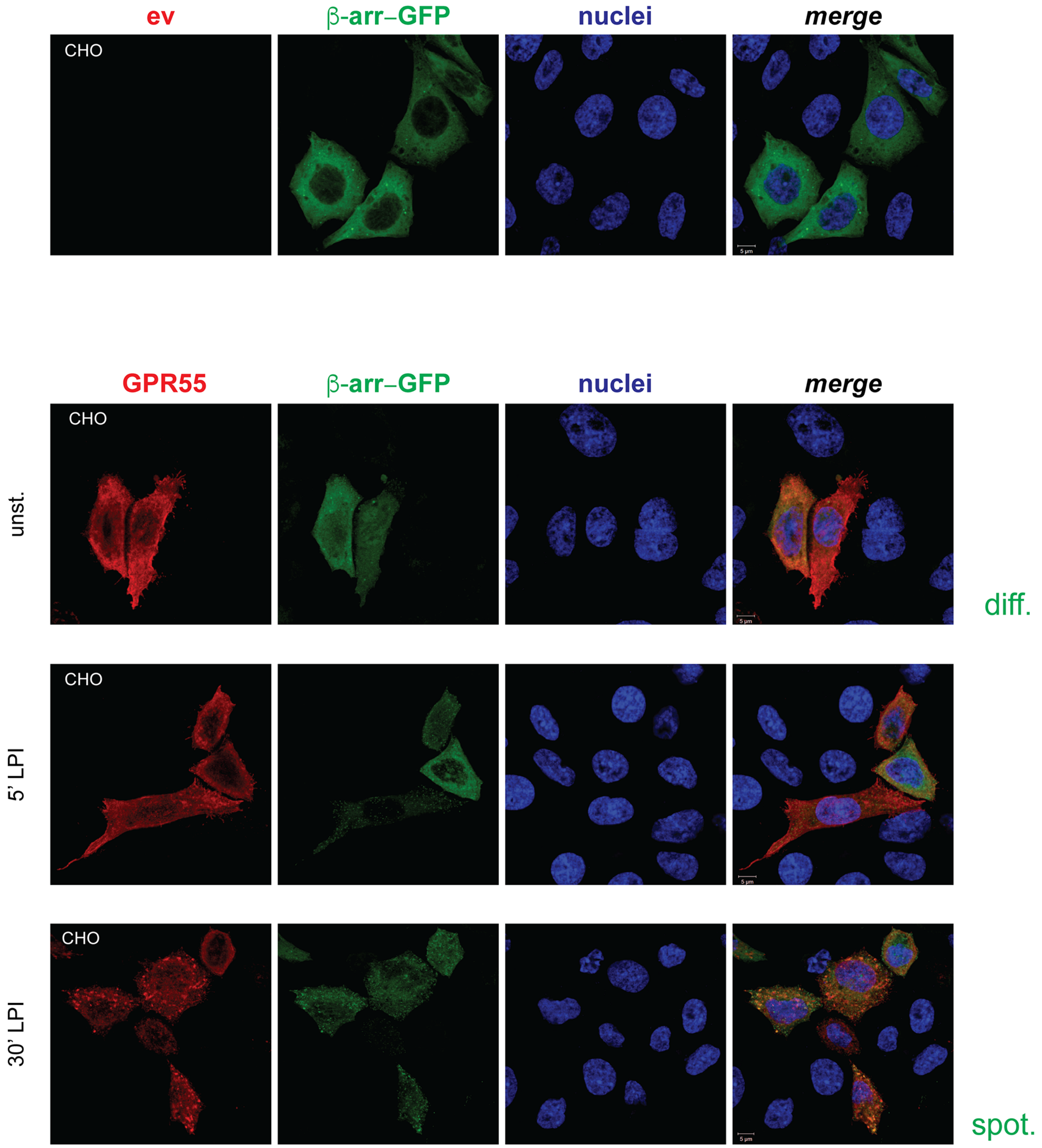

Supplementary Figure S5: Immunofluorescence analysis of β-arrestin–transfected CHO cells. Representative confocal images of CHO cells transfected with the empty vector (ev), or expressing HA-GPR55E (GPR55, red) and β-arrestin–GFP (green). β-Arrestin–GFP was uniformly distributed in the cell cytoplasm (diff.) of unstimulated cells (unst.) after 1 h serum deprivation. In HA-GPR55E-expressing cells, treatment for 5 min with 10 µM LPI resulted in plasma-membrane recruitment of β-arrestin–GFP, as shown by spotted distribution (spot.). Prolonged LPI treatment of up to 30 min induced HA-GPR55E and β-arrestin–GFP co-localization in endocytic structures. Hoechst staining for cell nuclei (blue) is also shown. Scale bar: 5 µm.

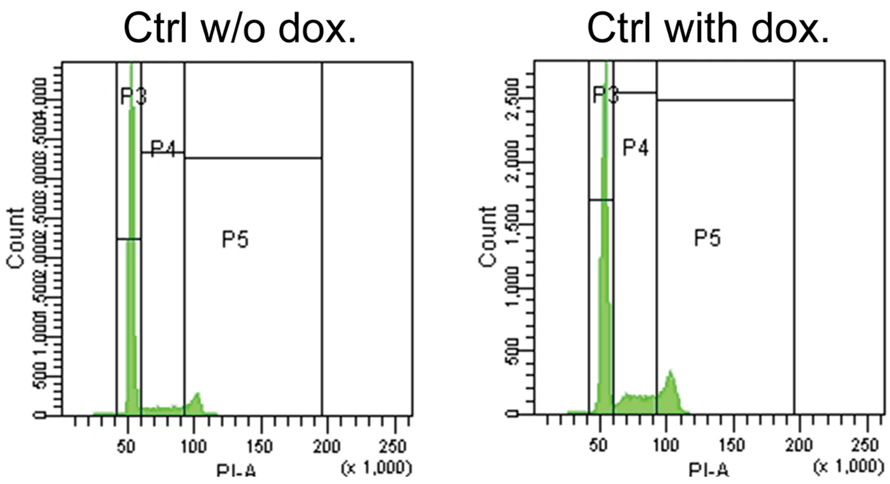

without 0.1 μM doxorubicin

cell-cycle phases	FACS gates	Ctrl (% tot)	P1-b (% tot)	Irr-b (% tot)	P1-b+str. (% tot)	Irr-b+str. (% tot)	Biotin+str. (% tot)	0.5 μM ML-191 (% tot)
G1	P3	75.8	76.3	75.1	75.8	75.2	72.6	77.4
S	P4	12.8	13.1	14.0	13.2	13.1	15.0	10.9
G2/M	P5	11.0	10.3	10.6	10.6	11.3	11.9	11.6

with0.1 μM doxorubicin

cell-cycle phases	FACS gates	Ctrl (% tot)	P1-b (% tot)	Irr-b (% tot)	P1-b+str. (% tot)	Irr-b+str. (% tot)	Biotin+str. (% tot)	0.5 μM ML-191 (% tot)
G1	P3	62.7	57.4	61.0	59.6	62.4	61.2	58.7
S	P4	18.8	21.1	19.1	18.5	17.8	19.6	18.2
G2/M	P5	18.3	21.0	19.5	21.5	19.5	18.8	22.8

Supplementary Figure S6: Cell-cycle distribution of peptide-treated EHEB cells. FACS analyses of EHEB cells untreated or pre-incubated with 0.5 µM ML-191, 1 µg/ml biotinylated peptide P1, or equimolar amounts of biotinylated irrelevant peptide (alone or with addition of 0.2 µM streptavidin). Biotin (0.8 µM) together with streptavidin (0.2 µM) was used as the negative control. The different agents, added *per se* or in combination with 0.1 µM doxorubicin, were added immediately after cell plating and replenished every 24 h. Ninety-six hours after plating, 1 $\times 10^6$ cells were harvested and processed for cell-cycle distribution. Representative data from three independent experiments are shown. The FACS histograms of EHEB cells untreated (Ctrl w/o dox.) and treated (Ctrl with dox.) with 0.1 µM doxorubicin, used as controls, are shown. The tables show the percentages of the total population (with 20,000 event counts) in the different cell phases, as derived from the FACS analysis.

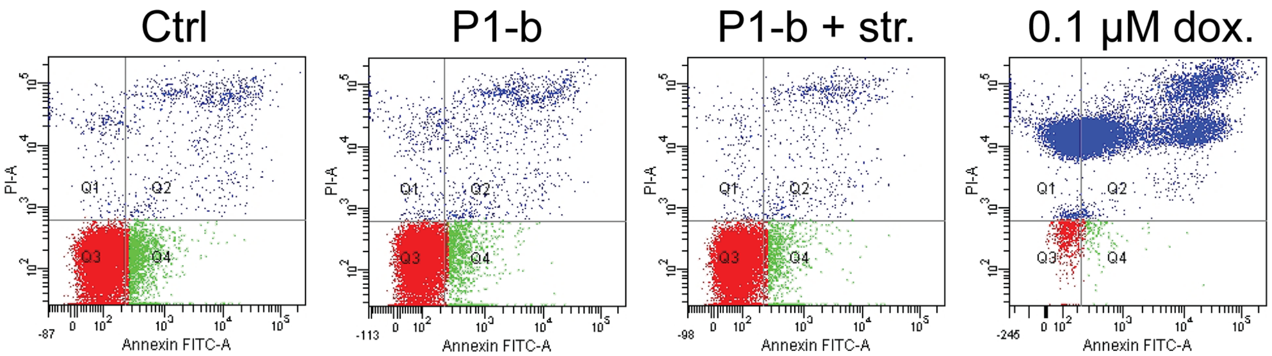

	FACS gates	Ctrl (% tot)	P1-b (% tot)	P1-b+str. (% tot)	0.1 μM doxorubicin (% tot)
**dead cells**	**Q1 / Q2**	1.3 / 4.4	1.7 / 6.1	1.1 / 3.7	38.1 / 57.8
**live cells**	**Q3 / Q4**	82.3 / 12.0	84.2 / 8.0	89.3 / 5.9	3.3 / 0.8
**annexin-V positive (dead)**	**Q2**	4.4	6.1	3.7	57.8
**annexin-V positive (live)**	**Q4**	12.0	8.0	5.9	0.8

Supplementary Figure S7: Annexin-V staining of peptide-treated EHEB cells. EHEB cells treated as in Supplementary Figure S6 were stained with fluorescent annexin V, using the human annexin V-FITC kit, and analyzed by FACS. A representative of two independent experiments is shown. The FACS dotplots of EHEB cells untreated (Ctrl), or treated with biotinylated-peptide P1 alone (P1-b) or with addition of 0.2 μM streptavidin (P1-b + str.), and or with 0.1 μM doxorubicin (dox.) are shown. The table shows the percentages of the total population (with 20,000 event counts) of annexin-V positive cells (live *vs* dead), as derived from FACS analysis.

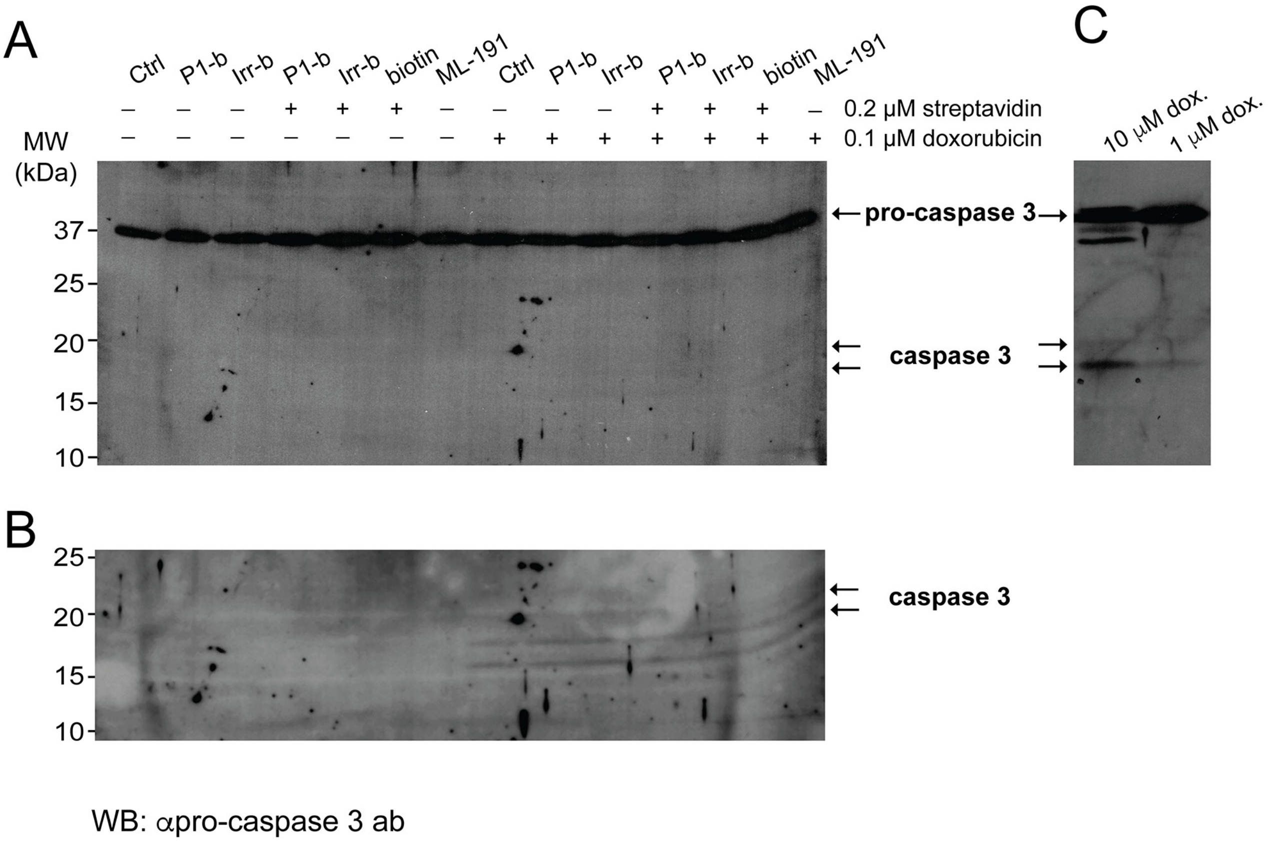

Supplementary Figure S8: Pro-caspase 3 activation in peptide-treated EHEB cells. EHEB cells were treated as in Supplementary Figure S6. **A.** One-hundred micrograms of cell lysates were evaluated by Western blotting for pro-caspase 3 activation. **B.** Longer exposure of the lower part of the membrane in panel A. **C.** Western blotting for pro-caspase 3 activation of doxorubicin-treated samples (1-10 μM dox.) is shown as positive control. MW, molecular weight standards.
